# Severe Traumatic Brain Injury in children—paradigm of decompressive craniectomy compared to a historic cohort

**DOI:** 10.1007/s00701-022-05171-4

**Published:** 2022-03-19

**Authors:** Vanessa Hubertus, Tobias Finger, Ricarda Drust, Sara Al Hakim, Andreas Schaumann, Matthias Schulz, Alexander Gratopp, Ulrich-Wilhelm Thomale

**Affiliations:** 1 Department of Neurosurgery, Charité-Universitätsmedizin Berlin, Corporate Member of Freie Universität Berlin, Humboldt-Universität zu Berlin, and Berlin Institute of Health, Berlin, Germany; 2 Pediatric Neurosurgery, Charité-Universitätsmedizin Berlin, Corporate Member of Freie Universität Berlin, Humboldt-Universität zu Berlin, and Berlin Institute of Health, Augustenburger Platz 1, 13353 Berlin, Germany; 3 Department of Pediatric Respiratory Medicine, Immunology and Critical Care Medicine, Charité-Universitätsmedizin Berlin, Corporate Member of Freie Universität Berlin, Humboldt-Universität zu Berlin, and Berlin Institute of Health, Berlin, Germany

**Keywords:** Pediatric traumatic brain injury, Severe TBI, Intracranial pressure, Decompressive craniectomy, Glasgow outcome score

## Abstract

**Purpose:**

Traumatic brain injury (TBI) is one of the leading causes of death and disability in children. Medical therapy remains limited, and decompressive craniectomy (DC) is an established rescue therapy in case of elevated intracranial pressure (ICP). Much discussion deals with clinical outcome after severe TBI treated with DC, while data on the pediatric population is rare. We report our experience of treating severe TBI in two different treatment setups at the same academic institution.

**Methods:**

Forty-eight patients (≤ 16 years) were hospitalized with severe TBI (GCS ≤ 8 points) between 2008 and 2018 in a pediatric intensive care unit (ICU) at a specialized tertiary pediatric care center. Data on treatment, clinical status, and outcome was retrospectively analyzed. Outcome data included Glasgow Outcome Scale (GOS) at 3-, 12-, and 36-month follow-up. Data was compared to a historic cohort with 53 pediatric severe TBI patients treated at the same institution in a neurointensive care unit between 1996 and 2007. Ethical approval was granted (EA2/076/21).

**Results:**

Between 2008 and 2018, 11 patients were treated with DC. Compared to the historic cohort, patients were younger and GCS was worse, while in-hospital mortality and clinical outcome remained similar. A trend towards more aggressive EVD placement and the internal paradigm change for treatment in a specialized pediatric ICU was observed.

**Conclusions:**

In children with severe TBI treated over two decades, clinical outcome was comparable and mostly favorable in two different treatment setups. Consequent therapy is warranted to maintain the positive potential for favorable outcome in children with severe TBI.

## Introduction

Traumatic brain injury (TBI) is one of the leading causes of death and disability in children aged ≤ 16 years in developed countries. Severe TBI occurs in approximately 5% of pediatric TBI cases and is defined as a Glasgow Coma Scale (GCS) ≤ 8 points. Thanks to the development of higher security standards in road transport, the incidence of severe TBI due to traffic accidents is globally decreasing [5, 20]. However, road traffic accidents and falls from height remain the most common causes of severe TBI in children [5, 26, 32, 35]. Medical options for the treatment of severe TBI remain limited and the Brain Trauma Foundation gives guidelines regarding medical therapy, therapy escalation, and the performance of emergency procedures to treat an elevated intracranial pressure (ICP) such as decompressive craniectomies (DC) [18, 19]. The performance of a DC is established as a rescue therapy in case of therapy refractory elevated intracranial pressure or a visible diffuse mass lesion in the CT scan [4, 19, 24]. However, much discussion deals with the clinical outcomes of severely brain-injured patients treated with or without DC [14, 16, 20, 30, 39]. Comprehensive data of clinical studies specialized on the treatment and related clinical outcome of severe TBI in the pediatric population are sparse [3, 10, 18, 19, 23–25, 43]. This lack in the literature is of special importance, as there are some distinct differences between pediatric and adult TBI. First, the pediatric skull and brain are more likely compressible, and fewer mass lesions and more white matter shear lesions occur. Second, diffuse brain swelling is much more frequent in pediatric than in adult TBI and the pathophysiological mechanisms leading to intracranial hypertension might differ between children and adults. Third, children are expected to recover better even from severe brain damages than adults [1, 17, 37, 44]. The Brain Trauma Foundation in their 2019 guidelines update shifted the awareness more towards the necessity for a specialized treatment of children with severe TBI [18]. Therefore, data of larger pediatric cohort studies and multicenter experiences over a longer time period are of high academic interest. With this study, we report our retrospective experience of treating children ≤ 16 years of age with severe TBI between 2008 and 2018 surgically or conservatively in our pediatric intensive care unit at a single academic neurosurgical institution in a specialized tertiary pediatric care center and compare the clinical presentation, therapy, and outcome up to 36 months after trauma with a historically reported cohort treated at a neurocritical care unit at the same institution from 1996 to 2007.

## Methods

Data sets of pediatric patients (≤ 16 years of age) consecutively hospitalized with severe TBI between 2008 and 2018 were retrospectively evaluated. The patients were treated by a pediatric neurosurgical and pediatric intensive care unit at a specialized tertiary pediatric care center in Germany. Forty-eight patients were identified with severe TBI, defined as GCS at admission ≤ 8 points. Exclusion criteria were moderate or mild TBI (GCS at admission > 8), non-accidental head injuries, and age at admission > 16 years. We collected demographic data (sex, age) as well as data regarding the trauma cause, clinical, and therapy data. Clinical data was retrospectively assessed through patient charts and radiologic imaging (CT/MRI), and included GCS, the presence of pupillary differences and light reaction, the description of the main brain pathology, the presence of a skull fracture or of intraventricular hemorrhage, the quantification of midline shift, and the necessity of intubation upon admission. Therapy data included the presence and length of an external ventricular drainage (EVD, placed by guided technique [38] or with ultrasonography if done parallel to DC) or of an intraparenchymal ICP probe (Raumedic®), the duration of ventilation therapy, the duration of hospitalization and stay in an intensive care unit (ICU), and surgical data. Surgical procedures included craniotomy with direct bone flap reimplantation, decompressive craniectomy (hemicraniectomy or bifrontal craniectomy) with duraplasty, and secondary cranioplasty. Outcome data was retrospectively assessed through the patient’s charts of the routine clinical follow-up at our pediatric institution. Loss to follow-up occurred when patients changed the clinical care center. The primary outcome parameter was set as the Glasgow Outcome Scale (GOS) at discharge and at 3- and 12-month follow-up. Secondary outcome parameters were defined as the development of posttraumatic hydrocephalus, ventriculo-peritoneal or subduro-peritoneal shunt necessity, surgical complications, and the necessity for re-operation as well as the mean duration of follow-up and GOS at 36 months. In a second step, we compared this patient population to a historic cohort with pediatric (≤ 16 years) severe TBI (*n* = 53) treated consecutively at the same institution between 1996 and 2007 but in a neurointensive care unit of an adult patient environment, as previously described. [37]

### Surgical and medical treatment

At first admission to the hospital, patients were treated in the emergency room by an interdisciplinary team comprising of a specialized pediatric traumatologist, pediatric anesthesiologist, and pediatric neurosurgeon. In case of moderate-severe TBI, a cranial CT scan was performed, when appropriate in combination with a polytrauma CT scan or X-ray of the extremities. Focal mass lesions were usually surgically evacuated in the emergency setting, either with craniotomy and bone flap reimplantation or in combination with DC, depending on the extension of the pathology in the initial CT scan and on clinical status. Patients thus treated initially with DC or with craniotomy for mass evacuation and being likely postoperatively unresponsive received an ICP probe or an EVD intraoperatively to continuously monitor ICP postoperatively. In the absence of a mass lesion but with the patient not responsive, ICP was also monitored via ICP probe or EVD. The placement of an EVD or an ICP probe without further surgical intervention was considered as conservative treatment in our management algorithm. ICP was treated according to the Brain Trauma Foundation guidelines for the treatment of severe pediatric TBI [18]. Secondary DC was performed in the case of (1) refractory and prolonged ICP elevation (> 20 mmHg) despite first-line ICP therapy and (2) in any case of early (< 48 h) uncontrollable ICP rise and/or beginning signs of brain herniation, following a previously described therapy algorithm [37]. All patients in this cohort (2008–2018) were medically treated and monitored on a specialized pediatric intensive care unit. In the historic cohort treated between 1996 and 2007, the medical treatment and monitoring was conducted on a neurocritical care unit, not exclusively specialized for pediatric patients.

### Decompressive craniectomy procedure

When mass lesions caused brain shift, an extensive decompressive fronto-temporo-parietal hemicraniectomy was performed on the side of the injury. When both sides were equally injured, or in the case of diffuse posttraumatic brain edema, a bilateral fronto-temporo-parietal craniectomy was performed while keeping the calvarial rim above the superior sagittal sinus. In all cases, the dura mater was opened and a duraplasty either using Lyomesh® (equine pericardium sheet) or periosteum was performed. Bone flaps were cryo-preserved and were usually reimplanted after 3–4 months during follow-up in case of the patient’s sufficient recovery [37].

### Clinical outcome data

Patients were monitored during their stay in the ICU by an interdisciplinary team of pediatric intensivists, pediatric anesthesiologists, pediatric neurosurgeons, and other consultants on demand. Medical and surgical complications, reoperations, development of posttraumatic hydrocephalus, and the necessity of a ventriculo-peritoneal or subduro-peritoneal shunt were assessed. Clinical outcome was evaluated at discharge and at 3-, 12-, and 36-month follow-up consecutively.

### Ethical statement

This study was conducted according to the ethical principles of medical research involving human subjects according to the Declaration of Helsinki. The clinical data were assessed retrospectively and anonymized for patients’ confidentiality. Written patient consent was waived due to the retrospective nature of the study. Ethical approval was granted by the institutional ethics board of the local ethics committee (EA2/076/21).

### Data management and statistical analysis

For data management and analysis, Microsoft Excel was utilized. Statistical analysis was performed using GraphPad Prism 9 (GraphPad Software, La Jolla, CA, USA). For statistical analysis, we used Student’s *T*-test or Kruskal–Wallis test as appropriate depending on Shapiro Wilk test for normal distribution. Kaplan–Meier survival curves were obtained, and differences in survival were tested for statistical significance using the log-rank test. Significance level was set at *p* < 0.05.

## Results

### Recent patient cohort (2008–2018)

#### Patient characteristics and clinical presentation

**Fig. 1:**
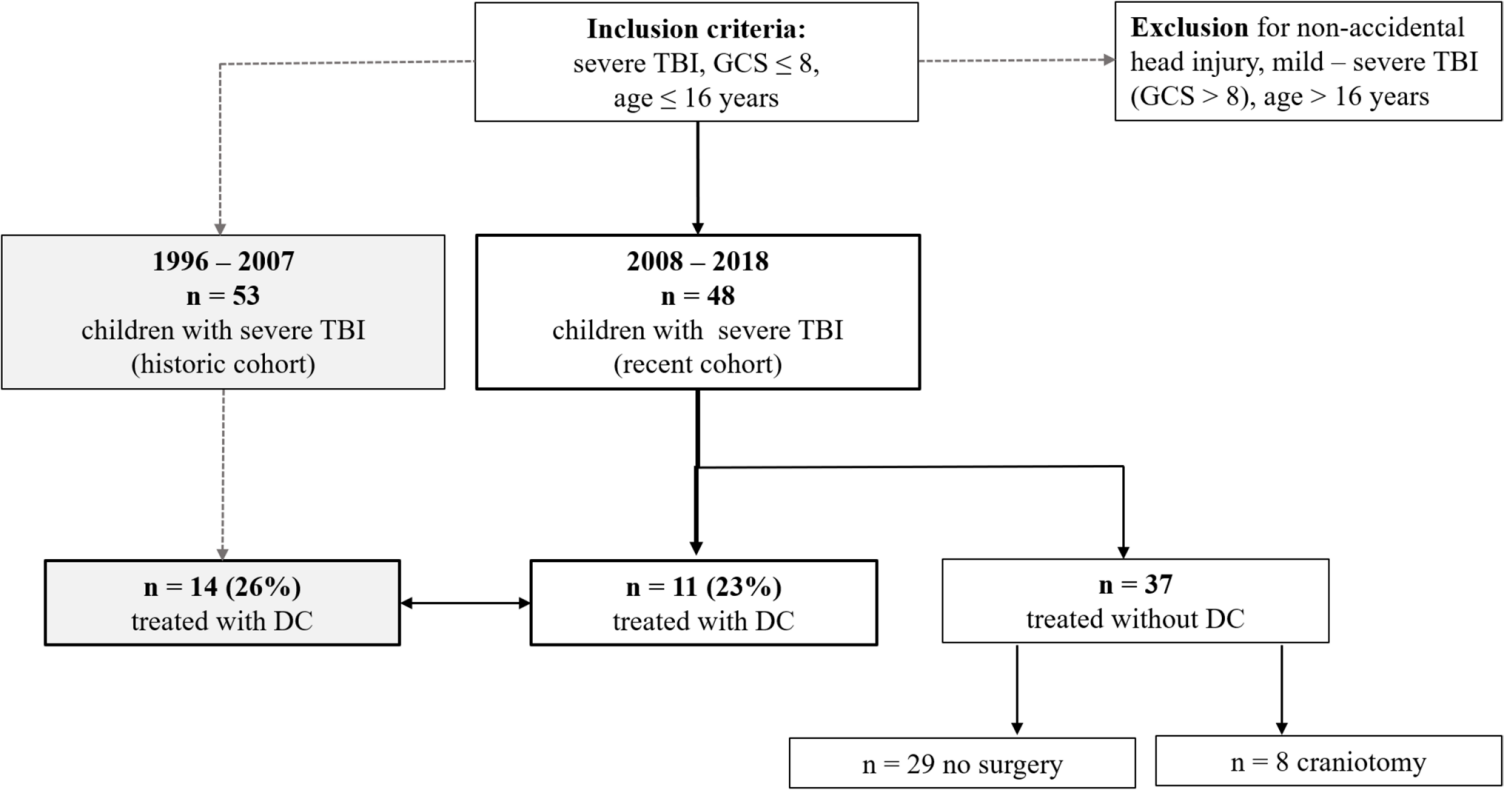
Illustration of study design and patient enrolment, comparison of the present to a historic cohort

**Fig. 2:**
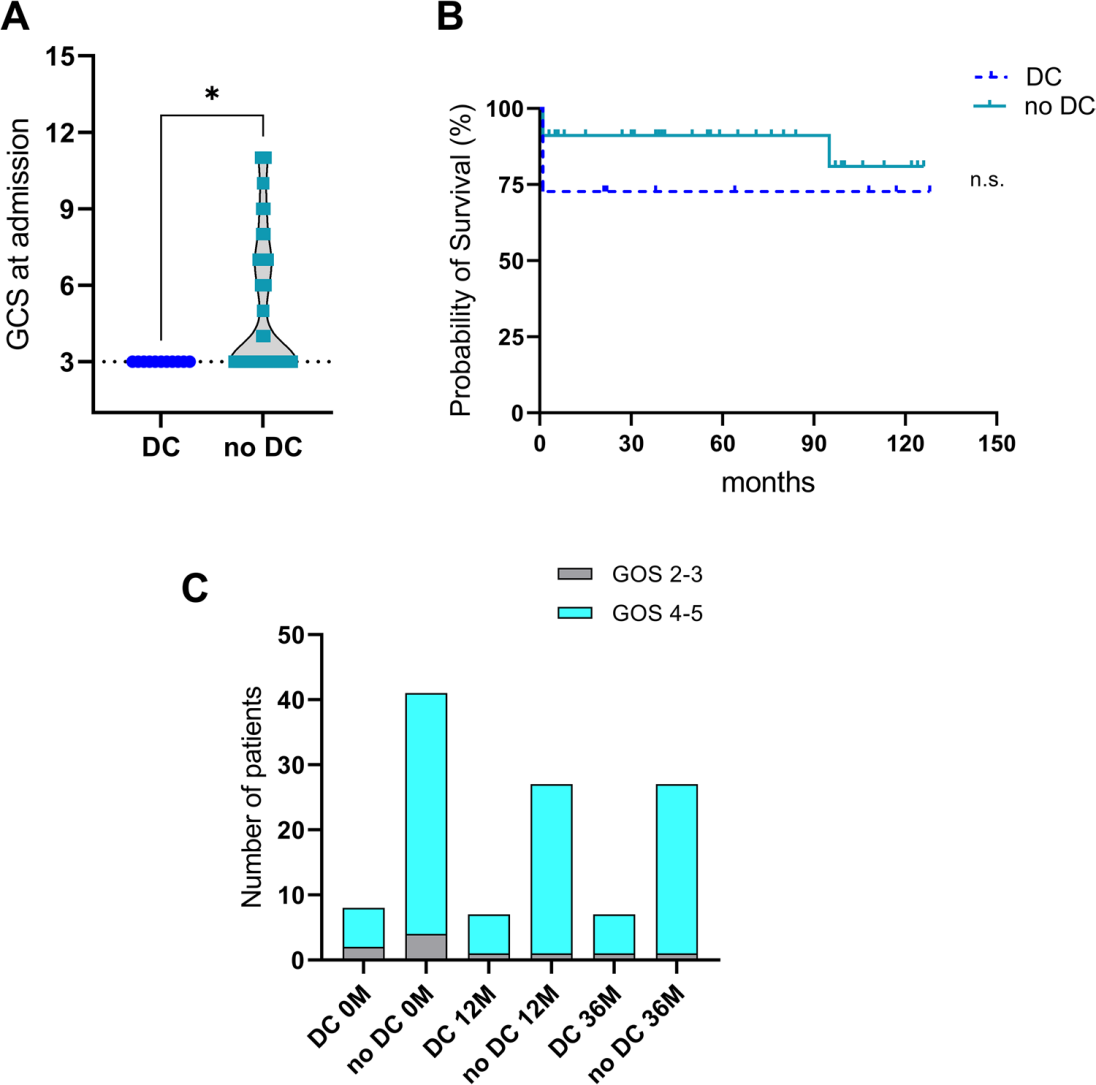
Comparison of the patients in the recent cohort (2008–2018) treated with or without decompressive craniectomy (DC vs. no DC). **a** Glasgow Coma Scale (GCS) at admission (*p* = *0.0365), **b** probability of survival (*p* = 0.2040), and **c** Glasgow Outcome Scale (GOS) during follow-up (favorable, GOS 4–5; non-favorable GOS 2–3), *p* = 0.2505 (n.s. = not significant) at 12 and 36 months

A total of 48 pediatric patients suffering from severe TBI (GCS ≤ 8 points) between 2008 and 2018 were enrolled in the study. All patients were admitted to a specialized pediatric ICU at a tertiary pediatric academic institution. The main causes of TBI were falls from height (52%), followed by traffic accidents (44%) and more rarely sports accidents (2%) or direct head trauma (2%). The median age at admission was 4 years (range 0–16), and 58% of the patients were male. Regarding trauma cause and related age span, patients with TBI due to falls from height were significantly younger than patients suffering from TBI due to traffic accidents (falls from height median age 4 years, range 0–16, traffic accidents median age 6 years, range 1–16, *p* = 0.0071). The one patient suffering from a sport accident was 15, and the one suffering from a direct hit to the head was 1 year old. Median GCS at admission was 3 points (range 3–8), and 44 patients (86%) were intubated at admission. In 31%, anisocoria was present, while 42% had impaired light reaction. In the radiological assessment of the initial CT scans, a skull fracture was present in 73% (35 cases) and intraventricular hemorrhage in 21% (10 cases). The main pathologies comprised in decreasing order of contusions (48%), subdural hematomas (SDH, 33%), epidural hematomas (EDH, 8%), diffuse edema in 8%, and intracerebral hematomas (ICH) in 2%. Midline shift was apparent in 56% and was measured at a median of 2 mm (range 0–12) (study enrollment, Fig. 1; patient’s characteristics at admission, Table 1, Fig. 2a).

**Table 1:** Patient’s characteristics and clinical presentation at admission. Comparison of the recent (2008–2018) to a historic cohort (1996–2007)

	All patients (1996–2018)	Recent cohort (2008–2018)	Historic cohort (1996–2007)	***p***value
*n*	**101**	**48**	**53**	n/a
Median age (years)	6 (0–16)	4 (0–16)	8 (0–16)	n/a
Sex, *n* (%)	Male 70 (69%) Female 31 (31%)	Male 28 (58%) Female 20 (42%)	Male 42 (79%) Female 11 (21%)	0.0867
Skull fracture, *n* (%)	65 (64%)	35 (73%)	30 (57%)	0.0999
Type of trauma, *n* (%)	Fall 43 (43%) Traffic 49 (48%) Other 9 (9%)	Fall 25 (52%) Traffic 21 (44%) Other 2 (4%)	Fall 18 (34%) Traffic 28 (53%) Other 7 (17%)	n/a
Main pathology, *n* (%)	EDH 23 (23%) SDH 25 (25%) Contusion 40 (39%) Other 13 (13%)	EDH 4 (8%) SDH 16 (33%) Contusion 23 (48%) Other 5 (10%)	EDH 19 (36%) SDH 9 (17%) Contusion 17 (32%) Other 8 (15%)	n/a
Median GCS at admission	5 (3–8)	3 (3–8)	6 (3–8)	n/a
Anisocoria, *n* (%)	31 (31%)	15 (31%)	16 (30%)	0.9119
Ventricular drainage, *n* (%)	68 (67%)	41 (85%)	27 (51%)	**0.0003
Median time of ventilation therapy (days)	7 (1–59)	8 (1–59)	5 (1–14)	n/a
Median ICU stay (days)	12 (1–77)	15 (1–77)	8 (3–20)	n/a
Median GOS at 3 months	5 (3–5)	5 (3–5)	4 (3–5)	n/a
Median GOS at 12 months	5 (3–5)	5 (3–5)	5 (4–5)	n/a

Values are given in total number with percentage or as median with total range, as appropriate. Statistical significance was tested between the recent and the historic cohorts by Student`s *T*-test or by Kruskal–Wallis test, depending on Shapiro Wilk test for normal distribution. * = *p* < 0.05, ** = *p* < 0.001, n/a = not applicable (as for the historic cohort treated without DC, only median and range or *n* and percentage was available). Abbreviations: *DC* decompressive craniectomy, *GCS* Glasgow Coma Scale, *GOS* Glasgow Outcome Scale, *EDH* epidural hematoma, *SDH* subdural hematoma

### Treatment and outcome data

In the recent cohort, 60% (29 cases) were conservatively treated. Craniotomy with direct bone flap reimplantation was performed in 17% (8 cases) and DC with duraplasty in 23% (11 cases). ICP measurement via EVD was performed in 85% (41 cases) and ICP monitoring via intraparenchymal probe (Raumedic®) in 38% (18 cases), as some patients received both (*n* = 12). The median time between admission to surgery was 1 day (range 0–6). The median duration of ventilation therapy was 8 days (range 1–59). The median duration of stay in an ICU was 15 days (range 1–77), and the overall hospitalization time was a median of 24 days (range 1–77). Three patients with severe TBI died after only 1 day in the ICU. Total in-hospital mortality was 15% (7 patients) (patient’s characteristics in this cohort: Table 2).

**Table 2:** Recent patient cohort (2008–2018) treated with or without decompressive craniectomy (DC). Patient’s characteristics and clinical presentation at admission and follow-up

	DC	No DC	***p*** value
*n* (% of all)	**11 (23%)**	**37 (77%)**	n/a
Median age (years)	5 (0–13)	3 (0–16)	0.6815
Sex (%)	Male 64%, female 36%	Male 57%, female 43%	0.6982
Type of trauma, *n* (%)	Fall 4 (36%), traffic 6 (55%), other 1 (9%)	Fall 21 (57%), traffic 15 (41%), other 1 (2%)	0.2444
Main pathology, *n* (%)	EDH 0 (0%), SDH 3 (27%), contusion 6 (55%), edema 2 (18%)	EDH 4 (11%) with surgery in 4 (100%), SDH 13 (35%) with surgery in 4 (31%), Contusion 17 (46%) with no surgery, Edema 3 (8%) with no surgery	0.1849
Skull fracture, *n* (%)	10 (91%)	25 (68%)	0.2456
Median GCS at admission	3 (3–3)	3 (3–8)	*0.0365
Anisocoria, *n* (%)	3 (27%)	12 (32%)	0.7600
Intact light reaction, *n* (%)	4 (36%)	24 (65%)	0.1622
Median midline shift (mm)	3 (0–9)	1 (0–12)	0.6248
Intubated, *n* (%)	11 (100%)	33 (89%)	0.5607
ICP probe, *n* (%)	EVD 10 (91%), Raumedic 1 (9%), both 8 (73%), none 0	EVD 31 (84%), Raumedic® 9 (24%), both 4 (11%), none 1 (3%)	0.6744 (EVD), **0.0010 (Raumedic®)
Median length of EVD (days)	14 (0–25)	5 (1 – 42)	0.4183
Median length of ventilation (days)	11 (0–59)	4 (0 – 36)	0.2525
Median ICU stay (days)	19 (1–77)	10 (1 – 51)	0.2192
Median hospital stay (days)	27 (1–77)	22 (1 – 67)	0.3008
Posttraumatic hydrocephalus, *n* (%)	4 (36%) VP-shunt 2, SDP-shunt 2	3 (8%) VP-shunt 0, SDP-shunt 3	*0.0392
In-hospital mortality, *n* (%)	3 (27%)	4 (11%)	0.3271
Median GOS at discharge	4 (1–5), n = 11	5 (1 – 5), n = 37	*0.0114
Median GOS at 12 months	4 (2–5), n = 7	5 (3 – 5), n = 24	0.2505
Median GOS at 36 months	4 (2–5), n = 7	5 (3 – 5), n = 24	0.2505

Values are given in total number with percentage or as median with total range, as appropriate. Statistical significance was tested between the cohort with DC and the cohort without DC by Student’s *T*-test or by Kruskal–Wallis test, depending on Shapiro–Wilk test for normal distribution. * = *p* < 0.05, ** = *p* < 0.001. Abbreviations: *DC* decompressive craniectomy, *GCS* Glasgow Coma Scale, *GOS* Glasgow Outcome Scale, *EDH* epidural hematoma, *SDH* subdural hematoma, *SDP* subduro-peritoneal shunt, *VP* ventriculo-peritoneal shunt

## Recent cohort treated with decompressive craniectomy (2008–2018)

### Etiology, clinical, and radiological findings

Of the eleven cases treated with DC, nine patients (82%) were treated with hemicraniectomy and two patients (18%) with bifrontal craniectomy. The main causes of trauma in this cohort were road traffic accidents (55%) and falls from heights (36%), whereas one patient suffered from a direct hit against the head. Radiological evaluation of the initial CT scans revealed the main pathologies in this cohort to be contusions (55%), SDH (27%), and diffuse edema (18%). Midline shift was existent in 73% (vs. 56% without DC), but the median midline shift was 3 mm (range 0–9), similar to that of patients treated without DC (median 2, range 0–12 mm). Marshall score was in the median 2 (range 2–5). Patients treated with DC in this cohort presented with a significantly more diminished GCS at admission (median 3 points, range 3–3 DC vs. median 3 points, range 3–8 without DC, *p* = 0.0216), and all DC patients were initially intubated (100%). Additionally, in patients treated with DC, pupillary light reaction was more often impaired (light reaction not intact in 64% (7 patients) DC vs. 29% (13 patients) without DC, *p* = 0.0313) while the number of patients presenting with anisocoria was similar (27% DC vs. 29% without DC).

### Medical and surgical treatment

A median of 2 days (range 0–6) passed between head injury and the performance of the DC. Nine out of eleven patients (82%) were treated with primary DC. Patients treated with DC received an EVD at a similar rate (91% DC vs. 80% without DC) and a higher rate of intraparenchymal ICP probe placements (Raumedic® in 82% DC vs. 20% without DC, *p* < 0.0001) as well as by tendency a longer EVD (14, range 0–25 DC vs. 5, range 1–18 days without DC) and Raumedic® duration (13, range 0–15 DC vs. 7, range 1–17 days without DC). Length of ventilation therapy (11 days, range 0–59 DC vs. 3 days, range 0–36 without DC, *p* = 0.2525), stay in an ICU (19 days, range 1–77 DC vs. 9 days, range 1–51 without DC, *p* = 0.2192), and overall hospitalization time (27 days, range 1–77 DC vs. 18 days, range 1–67 without DC, *p* = 0.3008) were non-significantly increased in patients treated with DC. Only one patient developed a surgical site infection (SSI) necessitating wound revision (9% of all patients treated with DC). Bone flap reimplantation during follow-up was performed in 73% (8 cases) at a median of 46 days (range 21–159) post trauma. In six patients (75%), bone flap reimplantation was performed during the initial hospital stay, while in two patients (25%), the reimplantation was performed in an additional hospital stay after 75 and 159 days. In only one of the patients receiving bone flap reimplantation during follow-up, a secondary explantation with implantation of a CAD plastic due to aseptic osteolysis was necessary at 2 years following trauma (patient’s characteristics in this cohort, Table 2).

### Outcome data in the recent cohort (2008–2018)

In all patients of the recent cohort, the median follow-up was 43 months (range 0–128, 45 months DC vs. 42 months without DC). In total, 15% (7 patients) developed a posttraumatic hydrocephalus, of whom 4 patients were treated with a ventriculo-peritoneal and 3 patients with a subduro-peritoneal shunt, in the median at 33 days (range 28–75) post trauma. The probability of developing a shunt-depending posttraumatic hydrocephalus was significantly higher in the patients treated with DC (DC 36%, without DC 8%, *p* = *0.0392). The probability of survival during follow-up did not differ in patients treated with or without DC. In-hospital mortality was 15% (*n* = 7) and did not differ significantly between patients treated with or without DC either (27%, *n* = 3 with DC vs. 11%, *n* = 4 without DC, *p* = 0.3271).

The median GOS at discharge in all patients was favorable (without DC 5 points, range 1–5, *n* = 45 vs. with DC 4 points, range 1–5, *n* = 11) and remained favorable at 12- and 36-month follow-up (with DC 4 points, range 2–5, *n* = 7, without DC 5 points, range 2–5, *n* = 24). Comparing favorable (GOS 4–5) and non-favorable outcome (GOS 2–3) at 12 and at 36 months, outcome did not differ significantly between patients treated with or without DC (*p* = 0.2505). Lost to follow-up at 12 and 36 months were 12% (*n* = 1) in the DC vs. 27% (*n* = 9) in the non-DC group (Table 2) (detailed clinical outcome data of the recent cohort: Fig. 2b–c**)**.

### Case examples of patients treated in the recent cohort (2008–2018)

**Fig. 3:**
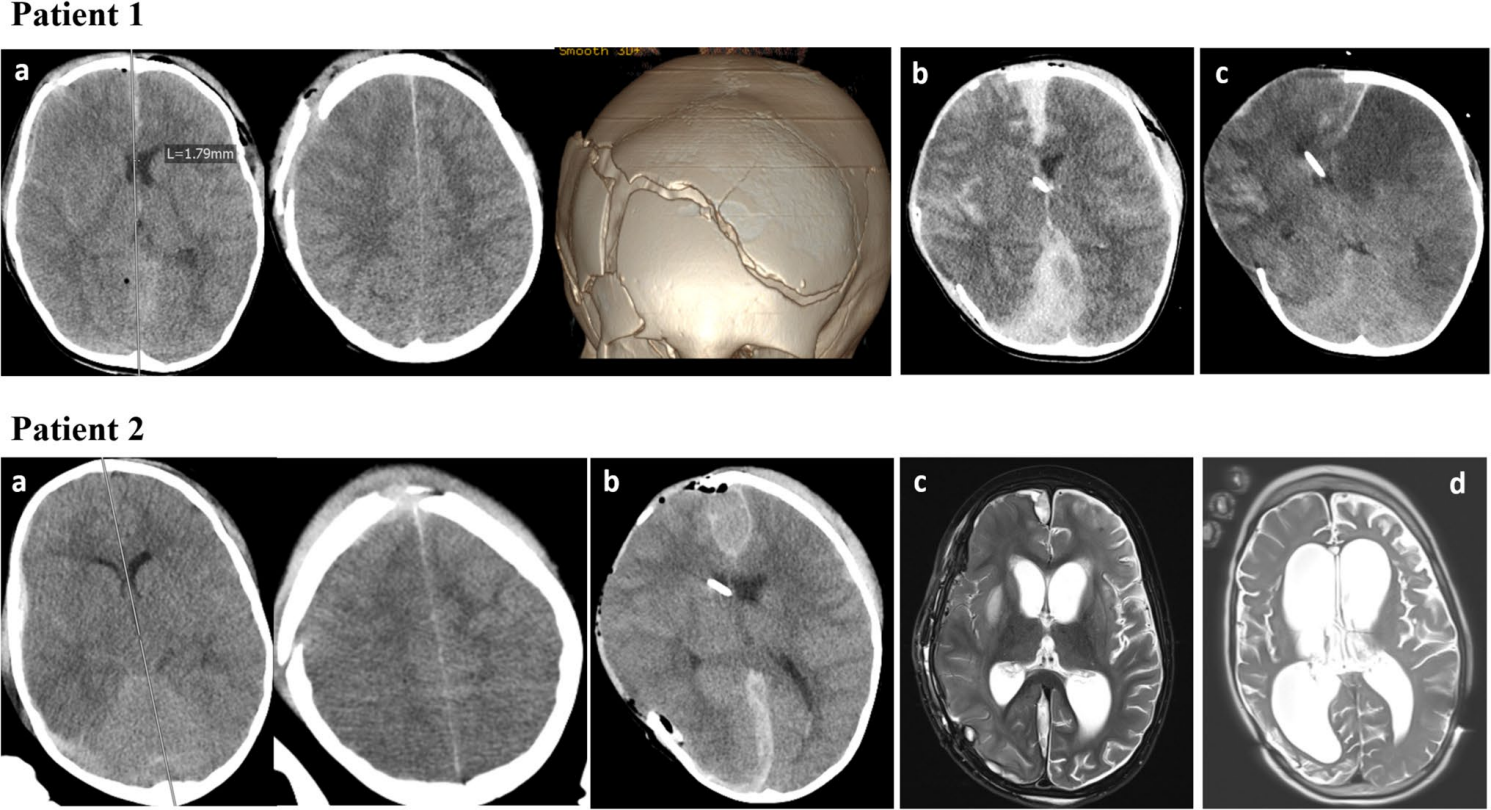
Exemplary cases of two children suffering from severe TBI and treated with decompressive craniectomy. Patient 1 was a 4-year-old boy suffering from a direct hit against the skull through the mirror of a past-driving car. The child presented with GCS 3, anisocoria, and non-intact light reaction. The initial CT scan (**a**) showed a complex frontal skull fracture and a diffuse TBI with a 2-mm midline shift. The child was treated with unilateral decompressive hemicraniectomy, and an EVD and an ICP probe were placed (postop scan: **b**). ICP was critically elevated throughout the postoperative course (ICP_max._ 40 mmHg), and the child presented with severe infarction and brain death at 14 days postop (**C**). Patient 2 is a 9-month-old boy who fell from a height and also presented with GCS 3, anisocoria, and non-intact light reaction. The CT scan (**a**) showed a right frontal skull fracture and a diffuse TBI without midline shift. The child was also treated with unilateral decompressive hemicraniectomy, and an EVD and an ICP-probe were placed. ICP_max_. was 21 mmHg (postop scan: **b**). Cranioplasty with reimplantation of the formerly removed bone flap was performed 21 days postop (**c**) without complications. The child showed brain hypotrophy in the follow-up scans (**d**, 2-year follow-up) and lives with respective disabilities (GOS 4)

In Fig. 3, we give two representative cases treated with DC between 2008 and 2018 at our institution. The first patient was a 4-year-old boy suffering from a direct hit against the skull through the back view mirror of a passing car. The child presented with GCS 3, anisocoria and non-intact light reaction in the ER. The initial CT scan (**a**) showed a complex frontal skull fracture and a diffuse TBI with a 2-mm midline shift. The child was treated with unilateral decompressive hemicraniectomy, and an EVD and an ICP probe were placed (postop scan: **b**). ICP was critically elevated throughout the postoperative course (post-DC ICP_max._ 40 mmHg) and the child presented with severe infarction and brain death at 14 days postop (**c**).

The second patient is a 9-month-old boy who fell from a height and presented with GCS 3, anisocoria, and non-intact light reaction. The CT scan (**a**) showed a right frontal skull fracture and a diffuse TBI without midline shift. The child was also treated with unilateral decompressive hemicraniectomy, and an EVD and an ICP-probe were placed. Post-DC ICP_max_ was 21 mmHg (postop scan: **b**). Cranioplasty with reimplantation of the formerly removed bone flap was performed 21 days postop (**c**) without complications. The child showed partial brain atrophy in the follow-up scans (**d**, 2-year follow-up) and survived with respective disabilities (GOS 4).

### Historical comparison of DC patients between the cohorts

#### Etiology, clinical, and radiological findings

**Fig. 4:**
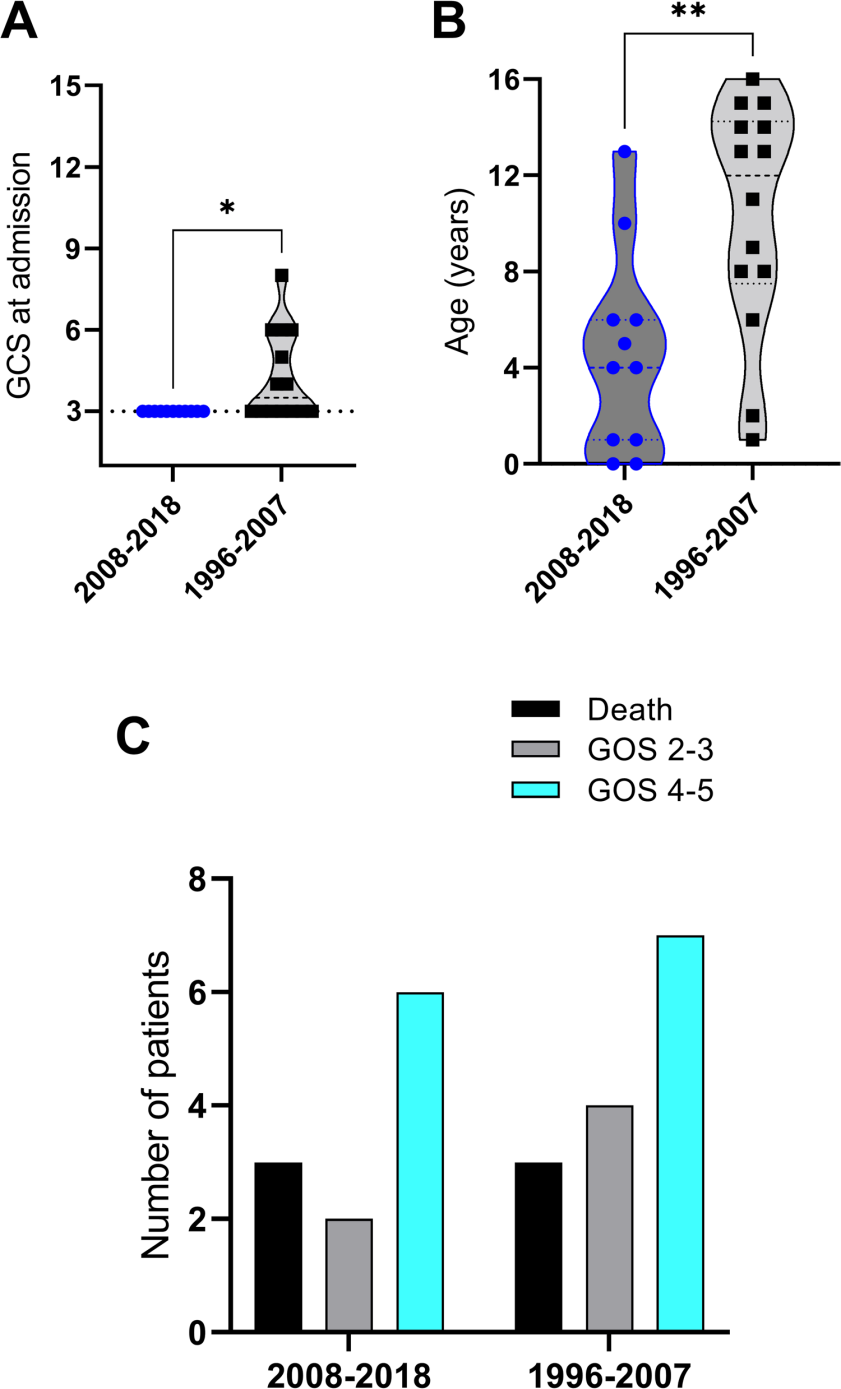
Comparison of the patients treated with decompressive craniectomy (DC) in the recent cohort (2008–2018) compared to the historic cohort (1996–2007). **a** Glasgow Coma Scale (GCS) at admission (*p* = *0.0163), **b** age at admission (*p* = *0.0043), **c** outcome data with Glasgow Outcome Scale (GOS) at discharge (favorable outcome GOS 4–5, non-favorable outcome GOS 2–3, death GOS 1) compared between patients treated in the recent (2008–2018) and in the historic cohort (1996–2007) with and without decompressive craniectomy (DC)

To describe the evolution of surgical decision-making and regarding the performance of DC in severe pediatric TBI at our institution, we compared the clinical and surgical data collected in the recent compared to a historic cohort [37]. Between 1996 and 2007, 14 out of 53 patients (26%) consecutively admitted to an adult neurocritical care unit with severe TBI (GCS ≤ 8 points) were treated with DC (current cohort: 24%) (comparison of clinical data at admission, Table 2, Fig. 4; comparison of surgical data, Table 3).

**Table 3:** Comparison of the recent cohort (2008–2018) vs. a historic cohort (1996–2007) treated with decompressive craniectomy (DC)

	All patients with DC (1996–2018)	Recent cohort with DC (2008–2018)	Historic cohort with DC (1996–2007)	*p* value
*n*	**25 (23%)**	**11 (20%)**	**14 (26%)**	n/a
Median age (years)	7 (0–16)	4 (0–13)	10 (0–16)	*0.0043
Sex, *n* (%)	Male 16 (64%) Female 9 (36%)	Male 7 (64%) Female 4 (36%)	Male 9 (64%) Female 5 (36%)	0.9746
Type of trauma, *n* (%)	Fall 8 (32%) Traffic 14 (56%) Other 3 (12%)	Fall 4 (36%) Traffic 6 (55%) Other 1 (9%)	Fall 4 (29%) Traffic 8 (57%) Other 2 (14%)	0.7215
Main pathology, *n* (%)	EDH 5 (20%) SDH 6 (24%) Contusion 10 (40%) Edema 4 (16%)	EDH 0 (0%) SDH 3 (27%) Contusion 6 (55%) Edema 2 (18%)	EDH 5 (36%) SDH 3 (21%) Contusion 4 (29%) Edema 2 (14%)	0.1094
Skull fracture, *n* (%)	18 (72%)	10 (91%)	8 (57%)	0.2443
Median GCS at admission	4 (3–8)	3 (3–3)	4 (3–8)	*0.0163
Anisocoria, *n* (%)	7 (28%)	3 (27%)	4 (28%)	0.9457
Median ICP_peak_ (mmHg)	29 (7–100)	25 (7–100)	32 (10–90)	0.9096
Median Marshall score	4 (2–5)	2 (2–5)	5 (5–5)	** < 0.0001
Type of DC, *n* (%)	Unilateral 15 (60%) Bilateral 10 (40%)	Unilateral 9 (82%) Bilateral 2 (18%)	Unilateral 6 (43%) Bilateral 8 (57%)	0.0508
Median time to DC (days)	2 (0–6)	2 (0–6)	2 (0–4)	0.1504
Median length of ventilation (days)	10 (0–59)	11 (0–59)	9 (5–19)	n/a
Median length of stay on an ICU (days)	20 (1–77)	19 (1–77)	20 (4–29)	n/a
In-hospital mortality, *n* (%)	6 (24%)	3 (27%)	3 (21%)	> 0.9999
Median GOS at discharge	4 (1–5), *n* = 25	4 (1–5), *n* = 11	4 (1–5), *n* = 14	0.9746
Median GOS at 3 months	4 (2–5), *n* = 18	4 (2–5), *n* = 7	4 (3–5), *n* = 11	n/a
Median GOS at 12 months	4 (2–5), *n* = 18	4 (2–5), *n* = 7	4 (3–5), *n* = 11	n/a

Values are given in total number with percentage or as median with total range, as appropriate. Statistical significance was tested between the recent and the historic cohorts by Student`s *T*-test or by Kruskal–Wallis test, depending on Shapiro Wilk test for normal distribution. * = *p* < 0.05, ** = *p* < 0.001, n/a = not applicable (as for the historic cohort DC, for some values only median and range or n and percentage was available). *EDH* epidural hematoma, *SDH* subdural hematoma, *GCS* Glasgow Coma Scale, *GOS* Glasgow Outcome Scale, *DC* decompressive craniectomy, *ICU* intensive care unit

The median age was significantly older in the historic cohort (median 10 years, range 0–16 vs. 4 years, range 0–13 recent cohort, *p* = *0.0043). Type of trauma, main pathology in the CT scan, and the occurrence of skull fractures showed non-significant differences (Table 3), but GCS at admission was significantly higher in the historic cohort (median 4, range 3–8 vs. 3, range 3–3, current cohort, *p* = *0.0163). The occurrence of anisocoria at admission was similar, as was ICP_peak_ (median 32 mmHg, range 10–90 historic vs. 25 mmHg, range 7–100, recent cohort). However, the trauma severity on the initial CT scan as measured by the Marshall Score was significantly worse in the historic cohort (median 5 points, range 5–5 vs. median 2 points, range 2–5, recent cohort, p** < 0.0001). Moreover, a significant trend towards more aggressive indication towards the primary treatment with DC was observed, with only 35% (5 out of 14 patients) in the historic cohort but 82% (9 out of 11 patients) treated with DC directly at admission (p* = 0.0419). In patients treated with primary DC in the current cohort, Marshall score was 5 points in 3/11 patients (27%), whereas in the historic cohort, Marshall score was 5 points in 14/14 patients (100%).

### Outcome data

In-hospital mortality did not differ significantly between cohorts (27% current vs. 21% historic cohort) and clinical outcome at discharge, and at 3 and 12 months post trauma was comparable with a median GOS of 4 points (range 3–5) in the historic cohort vs. 4 points (range 2–5) in the recent cohort, with an overall rate of patients lost to follow-up of 28% (Table 3).

## Discussion

To our knowledge, we report one of the largest retrospective cohort studies in children ≤ 16 years of age with severe TBI treated with or without decompressive craniectomy at a single academic pediatric institution.

Though TBI is a major cause of morbidity and mortality in children, only a few and mostly observational studies exist with a limited number of participants investigating the effect of DC on patient outcome in the pediatric population [10, 17, 25, 37]. One of the largest series so far was recently reported by Semenova et al. (2021), including 64 children with severe TBI receiving DC (out of 287 with severe TBI) [33].

According to the guidelines of the Brain Trauma Foundation, the performance of DC is considered as an effective manoeuver for rescue therapy in case of diffuse mass lesion and refractory ICP elevation under optimized medical care [18, 19]. Large randomized controlled trials in adults show the beneficial effect of DC on patient survival [6, 14, 28, 30]. As children are expected to recover better even from severe brain injuries compared to adults, they might profit from a more aggressive therapy algorithm, and the question of the most effective timing of DC in the paradigm of TBI therapy remains [8, 19, 24, 34, 37].

Several studies favor early DC and compare this paradigm to favorable patient outcome [2, 7, 8, 22, 31, 34]. In this study, DC was performed relatively early at 2 days (median 2, range 0–6 days) following trauma. In patients initially treated with DC, either any diffuse mass lesion was present in the initial CT scan and DC was performed to effectively treat ICP elevation or clinical signs of severe elevated ICP were present (anisocoria or absent light reaction). In the other patients, no unilateral mass lesion was present and therapy commenced with the placement of an EVD or an ICP probe (Raumedic®) followed by medical ICP treatment and DC was performed secondarily in case of refractory elevated ICP [18, 19, 37].

In relation to the consensus statement on the role of DC in the management of TBI by Hutchinson et al. (2019), the first consensus met the performance of a primary DC for mass lesion evacuation [13]. In our data, we observed a significant trend towards the indication for primary DC in this subgroup of patients (83% in the recent vs. 35% in the historic cohort). However, distinct differences existed in the management algorithm between our historic and recent cohorts: As the Marshall score was 5 points in 14/14 patients treated with DC in our historic cohort (100%), only 3/11 patients (27%) presented with a Marshall score of 5 points in our current cohort treated with DC. The other patients treated with DC in our study were treated with secondary DC, as to the second consensus by Hutchinson et al. due to refractory elevated ICP or as a second-tier therapy and the occurrence of secondary mass lesions and extensive brain swelling. However, the effect of secondary DC on outcome is not described as straightforward in the consensus statement, whereas results of different clinical studies favor the influence of early DC on patient outcome [6, 7, 13, 14].

As to the DC procedure, in this study an extensive decompressive fronto-temporo-parietal hemicraniectomy was performed on the more affected side of the injury unilaterally dominant injury. When both sides were equally injured, or in the case of diffuse posttraumatic brain edema, a bilateral fronto-temporo-parietal craniectomy at approximately 10–20 mm laterally of the superior sagittal sinus may be performed. In all cases, the dura mater was opened and duraplasty was performed. Extensive bony decompression is necessary for DC to decrease elevated ICP sufficiently and discontinuing the rigid dura mater is also beneficial for this effect [14, 15, 20, 28, 30]. The heterogeneity in surgical treatment is a known and much discussed limitation factor of TBI studies in the literature, as no standard surgical therapy recommendation for decompression surgery exists [13, 14, 33].

In this study, bone flaps were cryo-preserved and were reimplanted either before discharge or at 3–4 months during follow-up in case of the patient’s sufficient recovery [37]. In the present patient cohort, bone flap reimplantation was possible in all eight surviving patients. In only one of these patients, an aseptic bone necrosis necessitated secondary coverage by a computer-aided designed (CAD) heterologous bone implant at 2 years following injury. Therefore, we could not add to the body of literature discussing a negative effect on reimplantation of autologous bone implants after DC in children [12, 21, 27, 36].

A total of 101 patients were treated with severe TBI at a single academic institution between 1996 and 2018 in this study. Of these, a total of 25% (*n* = 25) were treated with DC. Although those patient’s GCS and clinical status at admission were significantly worse than of those patients treated without DC (GCS median 3 points in > 2008 and 4 points < 2008), in-hospital mortality did not differ significantly and median GOS up to 36-month follow-up was comparably favorable in both groups (GOS with DC in the median 4 points, without DC in the median 5 points). Hence, surviving children treated with or without DC showed to a high proportion either a good outcome or only minor disabilities. Thus, our data shows a trend towards an ameliorated clinical outcome of more severely injured children treated with DC, comparable to the outcome of lesser injured children in this cohort.

The data by Semenova et al. support the assumption that ICP is the main predictor of outcomes after severe TBI [6, 7, 33] and listed elevated ICP > 40 mmHg, low GCS < 6 points, non-intact pupillary status, and Marshall score of 3 as predictors for poorer outcome measured by the GOS at 6 months following TBI [33]. Our data supports the safety of DC in regard to clinical patient outcome regardless of a significantly poorer GCS at admission in the recent patient cohort compared to the historic cohort, with the GOS at 12 and 36 months remaining comparably favorable. Moreover, the Marshall score in our study did not influence in-hospital mortality or GOS at discharge and follow-up in children treated with DC. Regarding the patient cohort, in the study by Semenova et al. the patients were older and had a longer period between TBI and admission to hospital, and DC was exclusively performed secondary to refractory elevated ICP. In our cohort, DC was performed rather more consequently directly after admission in the presence of a mass lesion or clinical signs of elevated ICP (e.g., anisocoria, absent pupillary reaction), and diffuse swelling with a significant trend towards more aggressive indication in the recent (83%) than in the historic cohort (35%), potentially influencing patient outcome.

With the recent and the historic cohort together, this study covers an observation period of more than 20 years (1996–2008). Over this period, internal changes in the treatment paradigm of severely brain-injured children at our institution could be detected. As the Brain Trauma Foundation guidelines did not change substantially in regard to medical treatment algorithms, the 2019 update put more weight on the importance of treating children with severe TBI in a specialized pediatric ICU [17, 18]. Accordingly in our study, the historic cohort (1996–2007) was treated on a neurocritical care unit, next to adult patients with severe TBI. For the recent cohort (2008–2018), our internal treatment paradigm shifted and children with severe TBI were exclusively treated in a specialized pediatric ICU. However, mostly favorable clinical outcome and low in-hospital mortality remained similar, while a trend towards more aggressive EVD placement in the recent cohort was observed (*p* = **0.0003) due to enhanced technique for catheter placement in small ventricles [38]. Moreover, our data suggests a trend towards a lower GCS at admission in the recent cohort compared to the historic cohort (median GCS 3 points recent cohort vs. 6 points historic cohort in all patients admitted, median GCS 3 points recent cohort vs. 4 points historic cohort in patients treated with DC). As the outcome data remains comparably favorable in the recent cohort in patients treated with and without DC, an overall therapy improvement might be hypothesized.

One of the limitations of this study obviously is its retrospective design. Moreover, the lack of a more detailed discussion on medical treatments performed in the ICU is an additional limitation, as the pathophysiology is multifactorial and medical therapy alterations could affect the patient’s outcome additionally to the surgical therapy. Therefore, prospective studies including and comparing medical treatment data in detail are necessary. Furthermore, we described the necessity of a ventriculo-peritoneal or a subduro-peritoneal shunt in the patients post TBI, but did not analyze in detail the various factors potentially contributing to a posttraumatic hydrocephalus and thus leading to shunt dependency, which would merit a follow-up project due to its high complexity [11, 41].

However, with the long time covered by this study (1996–2008) and the resulting relatively large patient cohort, we add to the existing literature on the treatment of pediatric TBI patients with or without DC. Another potential drawback of this study is the heterogenous nature of the historic and the recent study cohort treated with DC, as the patients in the current cohort were significantly younger (median age 4 current vs. 10 years historic) and had a significantly worse GCS at admission (median 3, range 3–3 current vs. 4, range 3–8 historic), which might hold a potential bias. In addition to the GOS, further health-related quality of life (HRQOL) assessment may be performed in future studies. This is especially important when other long-lasting consequences for children with TBI with seemingly good recovery are discussed, such as neuropsychologic impairments, a higher incidence of depression, attention deficit hyperactivity disorder, or substance abuse. [2, 9, 15, 25, 29, 40, 42] Therefore, future studies with prospective follow-up and detailed HRQOL analysis of children suffering from TBI will deliver more detailed and relevant data.

## Conclusion

In total, 101 children were treated with severe TBI at our institution between 1996 and 2018. Decompressive craniectomy was performed relatively early following trauma (at a median 2 days) in a total of 25% (*n* = 25) of the patients. Internal paradigm differed between the historic and the recent observation periods towards the treatment in a specialized pediatric ICU, towards more aggressive indication for EVD placements and DC performed more often as primary treatment option while no significant differences in outcome between the cohorts could be detected. Although clinical status at admission was significantly worse in patients treated with DC in the recent cohort, in-hospital mortality did not significantly differ in patients treated with or without craniectomy, and clinical outcome was only slightly lower but still comparably favorable in patients treated with DC (median GOS 4 points), suggesting a trend towards an ameliorated clinical outcome in children treated with DC.

## Data Availability

The datasets supporting the conclusions of this article are included within the article and its additional file.

## Abbreviations

ANOVA: Analysis of variance
CAD: Computer-aided design
CT: Computed tomography
DC: Decompressive craniectomy
EDH: Epidural hematoma
EVD: External ventricular drainage
GCS: Glasgow Coma Scale
GOS: Glasgow Outcome Scale
HRQOL: Health-related quality of life
ICP: Intracranial pressure
ICU: Intensive care unit
SDH: Subdural hematoma
SSI: Surgical site infection
TBI: Traumatic brain injury
